# Longitudinal Functional Outcomes Among Survivors of Childhood Lower Extremity Osteosarcoma

**DOI:** 10.3390/cancers18111790

**Published:** 2026-05-29

**Authors:** Amy M. Berkman, Matthew D. Wogksch, Robyn E. Partin, Terry Wilson, Michael D. Neel, Michael W. Bishop, Najat C. Daw, Fariba Navid, Sara M. Federico, Deokumar Srivastava, Melissa M. Hudson, Kirsten K. Ness

**Affiliations:** 1 Department of Epidemiology and Cancer Control, St. Jude Children’s Research Hospital, 262 Danny Thomas Research Pl, Memphis, TN 38105, USA; 2 Rehabilitation Services, St. Jude Children’s Research Hospital, 262 Danny Thomas Research Pl, Memphis, TN 38105, USA; 3 Department of Surgery, University of Tennessee Health Science Center, 901 Madison Ave, Memphis, TN 38163, USA; 4 Department of Pediatrics, Arkansas Children’s Hospital, 1 Children’s Way Slot #512-10, Little Rock, AR 72202, USA; 5 Department of Pediatrics, The University of Texas MD Anderson Cancer Center, 1515 Holcombe Blvd, Houston, TX 77030, USA; 6 Cancer and Blood Disease Institute, Children’s Hospital Los Angeles, Department of Pediatrics, Keck School of Medicine, University of Southern California, 1975 Zonal Ave, Los Angeles, CA 90027, USA; 7 Department of Oncology, St. Jude Children’s Research Hospital, 262 Danny Thomas Research Pl, Memphis, TN 38105, USA; 8 Department of Biostatistics, St. Jude Children’s Research Hospital, 262 Danny Thomas Research Pl, Memphis, TN 38105, USA

**Keywords:** childhood cancer survivors, osteosarcoma, functional outcomes

## Abstract

**Purpose**: Survivors of childhood lower extremity and pelvic osteosarcoma have a high burden of functional, strength, and range of motion (ROM) impairment. However, longitudinal trajectories of these outcomes have not been well studied. **Methods**: This longitudinal study included patients enrolled on an institutional therapeutic protocol with lower extremity or pelvic osteosarcoma. Functional assessments (functional mobility assessment, FMA) were performed at diagnosis, prior to surgery, 10–12 weeks post-surgery, end of therapy, and 6, 18, and 48 months post-therapy. Generalized mixed models were used to evaluate differences in FMA scores, strength, and ROM across time points. **Results**: The study included 35 patients (mean age 13.1 ± 3.4 years) with osteosarcoma of the lower extremity (n = 34) and pelvis (n = 1). The majority underwent a limb salvage procedure (74.3%). FMA scores were lowest at the pre-therapy time point (least squared (LS) mean 27.2, standard error (SE) 2.2) and highest at the 48-month follow-up time point (LS mean 48.4, SE 2.4, *p*-trend < 0.01). A large proportion of participants had scores indicating functional impairment throughout the study period including 80% of participants at the 48-month follow-up. Strength and ROM scores demonstrated similar trends in improvement over time; however, they remained below normative scores at long-term follow-up. **Conclusions**: A high burden of impairment in function, strength, and ROM persists among survivors of childhood lower extremity and pelvic osteosarcoma from diagnosis through long-term follow-up. **Implications for Cancer Survivors**: A lifelong focus on mitigating impairments in function, strength, and ROM is needed among survivors of childhood pelvic and lower extremity osteosarcoma.

## Simple Summary

Survivors of childhood lower extremity osteosarcoma are at risk for functional, strength, and range of motion impairments. In the current study, functional, strength, and range of motion testing was performed on children and adolescents with lower extremity or pelvic osteosarcoma upon diagnosis, throughout treatment, and through 48 months post-treatment. While participants experienced improvements in some measures during the early post-treatment period, a large proportion had scores indicating functional impairment throughout the study period. These findings highlight that physical impairments begin early and persist well into survivorship for children with a history of lower extremity osteosarcoma. A focus on rehabilitation, even after the completion of treatment, may be important to help survivors maintain and improve long-term functional status.

## 1. Introduction

Osteosarcoma is the most common malignant childhood bone tumor [1]. The current 5-year survival rate for patients who present with localized disease is approximately 70% for those diagnosed prior to age 20 years [2]. This has led to a growing population of long-term survivors of childhood osteosarcoma, often with exposure to significant doses of multiagent chemotherapy and surgical intervention, including limb salvage procedures and amputation [3]. These treatment exposures are associated with long-term functional impairment, which may be particularly prevalent among survivors of lower extremity and pelvic osteosarcoma [3,4].

Prior studies evaluating functional outcomes in survivors of childhood lower extremity osteosarcoma have focused either on short-term post-surgical outcomes or longer-term outcomes, often years after treatment [5,6,7,8,9]. For example, among 117 childhood osteosarcoma survivors (86.3% lower extremity) with a median of 24 years post-diagnosis, more than half of survivors exhibited impaired mobility and aerobic function and more than one-third had impaired lower extremity endurance and strength, compared with controls [10]. Without longitudinal evaluation of functional outcomes, from the early post-surgical period through long-term follow-up, it remains unknown whether early functional improvements are associated with long-term physical functioning in survivors of childhood lower extremity osteosarcoma.

To better understand the trajectory of functional outcomes in survivors of childhood lower extremity osteosarcoma, we evaluated objective measures of function (functional mobility assessment, FMA; strength; range of motion, ROM) among survivors treated on an institutional therapeutic protocol at diagnosis, prior to surgery, 10–12 weeks post-surgery, end of therapy, 6 months post-therapy, 18 months post-therapy, and 48 months post-therapy. Additionally, in a subset of survivors, we evaluated function, strength, and ROM > 5 years post-therapy.

## 2. Methods

### 2.1. Study Population

Participants included in this longitudinal study were those with lower extremity or pelvic osteosarcoma enrolled on OS08—a study of bevacizumab, a humanized monoclonal antibody against vascular endothelial growth factor, in combination with chemotherapy (Supplemental Table S1) for treatment of osteosarcoma (ClinicalTrials.gov Identifier: NCT00667342) [11]. Eligible patients for OS08 had newly diagnosed high-grade osteosarcoma or malignant fibrous histiocytoma of bone and were ≤30 years of age at the time of diagnostic biopsy. Additional inclusion criteria included no prior history of chemotherapy or radiation treatment, Karnofsky score ≥50 or WHO/ECOG ≤2 for patients aged ≥16 years, Lansky score ≥50 for patients aged <16 years, and adequate renal, cardiac, hematologic, and hepatic function. Participants were enrolled from 2008 to 2012 and provided informed consent for this IRB-approved study. Those with major surgical procedures or non-healing wounds occurring within 28 days prior to enrollment were excluded as were those with known bleeding diathesis, platelet disorders, coagulopathy, thrombosis, cardiac disease or hypertension, proteinuria, central nervous system disease, gastrointestinal perforation, or abdominal fistulas.

### 2.2. Functional Outcomes

#### Functional Mobility Assessment

The FMA was used to measure physical function. This assessment was specifically validated on children and adolescents after surgical intervention for lower extremity sarcoma [12]. Objective measures in the FMA include the timed up and down stairs (TUDS) time, timed up and go (TUG) time, and the 9-Minute Walk-Run (9MWR). Heart rate (HR) and rate of perceived exertion (RPE) were measured during the TUDS, TUG, and 9MWR. Physiological cost index (PCI), a measure of walking efficiency, was measured during the 9MWR. Pain, use of assistive devices (brace, cane, and crutches), satisfaction with walking quality, and participation in work, school, and sports are also included in the FMA. The best functional outcome is indicated with a score of 70, the maximal obtainable score on the FMA [13]. Impairment was defined as scores below the median age- and sex-defined reference values in the general population. The FMA was performed at diagnosis, prior to surgery, 10–12 weeks post-surgery, end of therapy, 6 months post-therapy, 18 months post-therapy, and 48 months post-therapy. Time points for all assessments were chosen to align with on-therapy time milestones or transitions of care at our institution.

### 2.3. Strength and Range of Motion

Strength measurements of both the non-surgical and surgical extremity were obtained using a hand-held dynamometer. Knee flexion and extension isometric muscle strength was measured among those aged ≥4 years. Active ROM measurements of the ankle, knee, and hip of both the non-surgical and surgical extremity were obtained with a goniometer following the Norkin and White guidelines [14]. Ankle dorsiflexion and plantarflexion and knee and hip flexion ROM were measured in those aged ≥4 years. Strength was measured at 6, 18, and 48 months post-therapy and ROM was measured at each follow-up time point as well as at diagnosis, prior to surgery, 10–12 weeks post-surgery, and end of therapy. Impairments in strength and ROM were defined as z-scores ≥1.5 SDs below general population normative values.

### 2.4. Long-Term Follow-Up

A subset of survivors treated on OS08 were enrolled in the St. Jude Lifetime Cohort (SJLIFE), an institutional review board-approved study including adults aged ≥18 years at the time of evaluation and ≥5 years post-diagnosis that was established to characterize late health outcomes of childhood cancer survivors [15,16,17]. Similar strength and ROM assessments were performed at SJLIFE follow-up; however, functional performance measures were different as SJLIFE includes survivors of many childhood cancer types and is not limited to survivors of lower extremity malignancies. The timed up and go (TUG), physical performance test (PPT) in adults, and the Bruininks–Oseretsky test of motor proficiency (BOT) short form (children and adolescents) were completed. The FMA was not completed. The TUG is a measure of the time it takes to rise from a chair, walk 3 m, turn and walk back, and sit down and has been validated as a measure to quantify functional mobility [18]. The PPT objectively evaluates domains of physical function utilizing tests that simulate activities of daily living [19]. The BOT evaluates motor proficiency and incorporates measures of fine motor integration and precision, manual dexterity, balance, bilateral coordination, running speed and agility, upper limb coordination, and strength and provides an overall mobility score [20]. Impairment on these measures was defined as scores ≥1.5 standard deviations below SJLIFE population controls’ normative values.

### 2.5. Statistical Analysis

Descriptive statistics were used to characterize the study participants (Table 1). Outcomes were both continuous and categorical. Linear mixed-effects models were used to examine the difference in raw FMA scores at pre-therapy, week 10 (prior to surgery), weeks 20–22 (10–12 weeks post-surgery), end of therapy, and 6-month, 18-month, and 48-month follow-up (Table 2). The same linear mixed-effects modeling strategy was used for the continuous strength and ROM measures (Table 3 and Table 4). A standard linear mixed-effects model was created to evaluate changes in the outcome over time, with time modeled as a fixed effect and participant ID included as a random effect to account for within-subject correlation. A first-order autoregressive covariance structure was specified to model declining correlation between repeated measurements as the time interval increased. A simple model was chosen due to a low participant number. The Akaike Information Criteria (AIC) and Bayesian Information Criteria (BIC) were inspected for three variance covariance structures in each model. These were unstructured, first-order autoregressive variance covariance, and compound symmetry. The first-order autoregressive structure was similar to compound symmetry and much lower than unstructured. The first-order auto regressive structure was used in the final models due to the correlation between each time point potentially decaying over time (time from surgery). Frequency of impairment for each outcome is also presented at all time points within the same tables. Stratified analyses were performed for tumor laterality, tumor location (ilium or femur vs. tibia or fibula), and surgery type (amputation vs. limb salvage), with tumor location and surgery type done as supplementary analyses. Additionally, to describe function in long-term survivors of osteosarcoma, a univariate analysis using age- and sex-specific z-scores based on community control performance measures was performed on participants who became 5+ year survivors and enrolled in the St. Jude Lifetime cohort (n = 14). Data from the long-term analysis are presented as continuous z-scores and percent impaired. Impairment was defined as z-scores ≤−1.5 standard deviations. Data were analyzed with SAS version 9.4 (SAS institute, Cary, NC, USA).

## 3. Results

### 3.1. Population Characteristics

**Figure 1:**
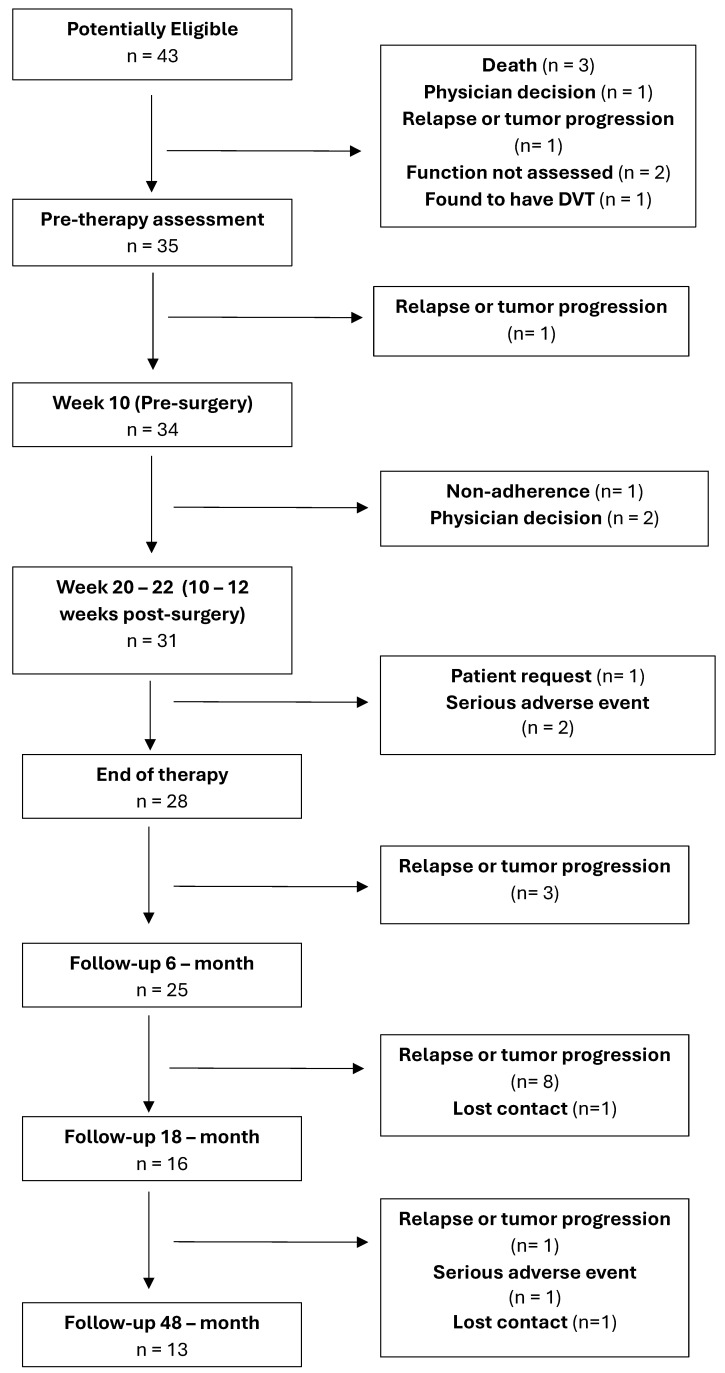
Consort diagram of participants enrolled on OS08 that underwent functional assessment.

Among the 43 osteosarcoma patients eligible at baseline, 35 underwent pre-therapy functional assessment (Figure 1), 34 had functional testing prior to surgery, 31 had testing ~12 weeks post-surgery, and 28 had testing at the end of therapy. In the follow-up period, 25, 16, and 13 patients underwent functional testing at 6-month, 18-month, and 48-month follow-up, respectively. Fourteen survivors enrolled in SJLIFE and had long-term functional outcomes assessment. Reasons for attrition from pre-therapy to 48-month follow-up were relapse or tumor progression (n = 13), serious adverse event (n = 3), physician decision (n = 2), lost contact (n = 2), patient request (n = 1), and non-adherence (n = 1). Forty six percent of participants were female, 28.6% were Black, and 57.1% were White (Table 1). The mean age at diagnosis was 13.1 years (standard deviation (SD) 3.4) and the majority of participants (58.8%) were diagnosed at stage IIB. Femoral tumors were the most common tumor location followed by tibial tumors. The majority of participants underwent a limb salvage procedure (74.3%) and 11.4% underwent above-the-knee amputation. All enrolled participants were treated with bevacizumab, cisplatin, dexamethasone, doxorubicin, and high-dose methotrexate and 25.7% of patients with metastatic disease at diagnosis also received etoposide and ifosfamide. Over the course of the study period, 40% of participants were hospitalized for infection (25.7% with one hospitalization and 14.3% with multiple hospitalizations) and over one-third were hospitalized for treatment toxicity (22.9% with one hospitalization and 14.2% with multiple hospitalizations).

**Table 1:** Participant characteristics.

	Participants (n = 35)
**Sex, n (%)**	
Female	16 (45.7)
Male	19 (54.3)
**Race, n (%)**	
Black	10 (28.6)
White	20 (57.1)
Other	5 (14.3)
**Age at diagnosis (years), mean (SD)**	13.1 (3.4)
**Tumor stage, n (%)**	
IIA	5 (14.7)
IIB	20 (58.8)
III	10 (26.5)
**Tumor location, n (%)**	
Femur, left	12 (32.4)
Femur, right	9 (26.6)
Fibula, left	1 (2.9)
Fibula, right	1 (2.9)
Tibia, left	5 (14.7)
Tibia, right	6 (17.6)
Iliac, right	1 (2.9)
**Local control, n (%)**	
Amputation, above knee	4 (11.4)
Amputation, below knee	3 (8.5)
Amputation, hemipelvectomy	1 (2.9)
Limb salvage procedure	26 (74.3)
Resection, tumor	1 (2.9)
**Thoracotomies, n (%)**	
None	28 (80.0)
One	3 (8.6)
Two	2 (5.7)
Three	1 (2.9)
Four	0 (0.0)
Five	1 (2.9)
**Endoprosthesis revision, n (%)**	2 (5.7)
**Endoprosthesis removal and replacement, n (%)**	2 (5.7)
**Wound debridement, n (%)**	
None	30 (85.7)
One	4 (11.4)
Two	1 (2.9)
**Hospitalized for infection, n (%)**	
None	21 (60.0)
One	9 (25.7)
Multiple	5 (14.3)
**Hospitalized for fever, n (%)**	
None	9 (25.7)
One	13 (37.2)
Multiple	13 (37.1)
**Hospitalized for pain, n (%)**	
None	30 (85.7)
One	4 (11.4)
Multiple	1 (2.9)
**Hospitalized for treatment toxicity, n (%)**	
None	22 (62.9)
One	8 (22.9)
Multiple	5 (14.2)

### 3.2. Functional Assessments

Least squared mean scores of the FMA test over time are shown in Table 2. Participants’ scores were lowest at the pre-therapy time point and highest at 48 months post-therapy (*p*-trend < 0.01). The least squared mean score on the FMA at pre-therapy was 27.2 (standard error (SE) 2.2) with least squared mean scores consistently increasing across time points to reach 48.4 (SE 2.4) at the 48-month time point. Scores were level from pre-surgery to 10–12 weeks post-surgery, and a large proportion of participants had scores indicating functional impairment at each time point. Eighty-seven percent of participants demonstrated impairment pre-therapy and 93.5%, 100%, 92.6%, 88%, 68.8% and 80% of those that completed testing demonstrated impairment at the pre-surgery (week 10), 10–12 weeks post-surgery, end of therapy, 6 months post-therapy, 18 months post-therapy, and 48 months post-therapy time points, respectively. When evaluating function by surgery type and tumor location, there were similar trends in FMA scores over time (Supplemental Table S2). There were differences in the proportion of survivors with impairment within these groupings, with 33.3% of those with amputation demonstrating impairment at the 48-month time point compared to 70.0% with limb salvage surgery, and 87.5% and 20.0% demonstrating impairment among those with a tumor in the iliac or femur and those with a tumor in the tibia or fibula, respectively.

**Table 2:** Mean (SE) and percent impairment of total functional mobility assessment score based on age and sex median normative scores.

	Pre-Therapy	Week 10	Weeks 20–22	End of Therapy	6 Months	18 Months	48 Months	*p*-Trend
**FMA score, LS mean (SE)**	27.2 (2.2)	30.6 (2.2)	30.0 (2.5)	35.4 (2.3)	40.8 (2.4)	46.3 (3.1)	48.4 (2.4)	**<0.001**
Impaired, n (%)	27 (87.1)	29 (93.5)	23 (100.0)	25 (92.6)	22 (88.0)	11 (68.8)	8 (80.0)	
Not impaired, n (%)	4 (12.9)	2 (6.5)	0 (0.0)	2 (7.4)	3 (12.0)	5 (31.2)	2 (20.0)	
Not completed	4	3	8	1	0	0	3	

FMA = functional mobility assessment; LS = least squared; SE = standard error. Bold indicates statistical significance.

### 3.3. Strength

For those with a right-sided tumor, affected quadriceps strength significantly improved from 6 months to 48 months post-therapy (*p* = 0.04, Table 3). While not reaching statistical significance, there was a trend towards improvement in least squared mean z-scores for unaffected quadriceps strength with z-scores of 1.69 (SE 0.62) and 2.76 (SE 0.82) at the 6-month and 48-month post-therapy time points, respectively (*p* = 0.08). Unaffected ankle dorsiflexion strength also improved over the course of follow-up, though this did not reach statistical significance (*p* = 0.07).

**Table 3:** Age- and sex-specific z-scores (least squared mean and SE) and percent of impairment (below −1.5 SEs) for isometric knee extension and ankle dorsiflexion strength measures.

	6 Months	18 Months	48 Months	*p*-Trend
**Left-Sided Tumor**	n = 14	n = 12	n = 10	
**Affected Quadriceps, LS mean (SE)**	−0.18 (0.56)	−0.17 (0.59)	0.59 (0.58)	0.156
Impaired, n (%)	3 (27.3)	1 (12.4)	2 (22.2)	
Missing	3	4	1	
**Unaffected Quadriceps, LS mean (SE)**	3.17 (0.61)	4.21 (0.64)	4.51 (0.69)	0.146
Impaired, n (%)	0 (0.0)	0 (0.0)	0 (0.0)	
Missing	0	0	2	
**Affected Ankle Dorsiflexion, LS mean (SE)**	−0.06 (0.53)	0.58 (0.55)	0.74 (0.57)	0.339
Impaired, n (%)	3 (27.3)	3 (30.0)	1 (11.1)	
Missing	3	2	1	
**Unaffected Ankle Dorsiflexion, LS mean (SE)**	2.41 (0.51)	3.11 (0.53)	2.64 (0.60)	0.367
Impaired, n (%)	0 (0.0)	0 (0.0)	0 (0.0)	
Missing	0	0	1	
**Right-Sided Tumor**	n = 11	n = 4	n = 3	
**Unaffected Quadriceps, LS mean (SE)**	1.69 (0.62)	1.20 (0.72)	2.76 (0.82)	0.079
Impaired, n (%)	0 (0.0)	0 (0.0)	0 (0.0)	
Missing	1	0	0	
**Affected Quadriceps, LS mean (SE)**	−1.09 (0.42)	0.65 (0.43)	3.81 (0.45)	**0.040**
Impaired, n (%)	2 (33.3)	0 (0.0)	1 (33.3)	
Missing	5	2		
**Unaffected Ankle Dorsiflexion, LS mean (SE)**	0.58 (0.68)	0.40 (0.88)	3.05 (1.05)	0.071
Impaired, n (%)	2 (20.0)	0 (0.0)	0 (0.0)	
Missing	1	0	0	
**Affected Ankle Dorsiflexion, LS mean (SE)**	−1.07 (0.67)	−0.37 (1.88)	−2.49 (1.88)	N/A ^a^
Impaired, n (%)	4 (50.0)	0 (0.0)	1 (100.0)	
Missing	3	3	2	

LS = least squared, SE = standard error; ^a^ *p*-value could not be estimated. Bold indicates statistical significance.

For those with a left-sided tumor, unaffected quadriceps strength remained similar from 6 months to 48 months post-treatment. Affected quadriceps strength demonstrated a trend towards improvement over time with least squared mean z-scores of −0.18 (SE 0.56), −0.17 (SE 0.59), and 0.59 (SE 0.58) at 6 months, 18 months, and 48 months, respectively (*p* = 0.16). However, the proportion of those with impairment in affected-side strength remained similar with 27.3%, 12.4%, and 22.2% demonstrating impairment at 6 months, 18 months, and 48 months post-therapy, respectively. Affected ankle dorsiflexion strength least squared z-scores also showed a trend towards improvement over the follow-up period, without reaching statistical significance (*p* = 0.34). There were no statistically significant trends in strength over time by surgery type or tumor location (Supplemental Table S3).

### 3.4. Range of Motion

Overall, survivors had ROM scores well below population normative values both throughout treatment and at follow-up (Table 4). Those with left-sided tumors had significant improvement in ROM scores from pre-therapy to 48-month follow-up in unaffected ankle plantarflexion and affected knee flexion and significant worsening in unaffected knee flexion. For those with right-sided tumors, affected ankle plantarflexion scores significantly improved from pre-therapy to 48-month follow-up. Despite improvement over time in some measures of ROM, a large proportion of survivors had multi-joint impairment in both affected and unaffected extremity ROM measures throughout treatment and follow-up. For example, among those with left-sided tumors who completed 48-month ROM testing, 60% and 40% had impaired affected and unaffected knee flexion, respectively. For those with right-sided tumors, 50% and 33% had impaired affected and unaffected knee flexion at 48-month follow-up, respectively. Results were similar when considering ROM outcomes by surgery type and tumor location, with scores well below population normative values at nearly all time points tested across surgery and tumor location categories and with no clear trends towards improvement (Supplemental Table S4).

**Table 4:** Age- and sex-specific z-scores (least squared mean and SE) and percent of impairment (below −1.5 SEs) for knee and ankle active range of motion.

	Pre-Therapy	Week 10	Weeks 20–22	End of Therapy	6 Months	18 Months	48 Months	*p*-Trend
**Left-Sided Tumor**	n = 18	n = 17	n = 16	n = 15	n = 14	n = 12	n = 10	
**Affected Ankle Dorsiflexion, LS mean (SE)**	−3.58 (0.44)	−3.32 (0.46)	−4.32 (0.46)	−4.54 (0.48)	−3.76 (0.47)	−3.52 (0.52)	−3.48 (0.54)	0.107
Impairment, n (%)	14 (93.3)	12 (92.3)	11 (84.6)	13 (100.0)	13 (100.0)	8 (80.0)	9 (100.0)	
Missing, n	3	4	3	2	1	2	1	
**Unaffected Ankle Dorsiflexion, LS mean (SE)**	−2.61 (0.25)	−3.02 (0.25)	−2.97 (0.25)	−3.11 (0.26)	−3.17 (0.27)	−3.06 (0.28)	−3.32 (0.30)	0.594
Impairment, n (%)	13 (76.5)	14 (82.4)	15 (93.8)	15 (100.0)	13 (92.9)	12 (100.0)	10 (100.0)	
Missing, n	1	0	0	0	0	0	0	
**Affected Ankle Plantarflexion, LS mean (SE)**	−2.04 (0.40)	−1.88 (0.43)	−2.18 (0.43)	−1.26 (0.43)	−1.34 (0.43)	−1.77 (0.49)	−0.45 (0.51)	0.146
Impairment, n (%)	10 (66.7)	9 (69.2)	9 (69.2)	5 (38.5)	6 (46.2)	6 (60.0)	2 (22.2)	
Missing, n	3	4	3	2	1	2	1	
**Unaffected Ankle Plantarflexion, LS mean (SE)**	−1.17 (0.28)	−1.55 (0.29)	−1.46 (0.29)	−0.63 (0.30)	−0.99 (0.31)	−1.20 (0.33)	−0.28 (0.35)	**0.028**
Impairment, n (%)	9 (52.9)	8 (47.1)	8 (50.0)	6 (40.0)	5 (35.7)	3 (25.0)	2 (20.0)	
Missing, n	1	0	0	0	0	0	0	
**Affected Knee Flexion, LS mean (SE)**	−6.41 (1.56)	−1.76 (1.39)	−8.14 (1.44)	−5.39 (1.51)	−3.03 (1.45)	−3.60 (1.57)	−4.23 (1.63)	**0.022**
Impairment, n (%)	7 (63.6)	4 (28.6)	9 (69.2)	9 (75.0)	8 (61.5)	7 (63.6)	6 (60.0)	
Missing, n	7	3	3	3	1	1	0	
**Unaffected Knee Flexion, LS mean (SE)**	0.50 (0.34)	1.08 (0.34)	0.91 (0.34)	1.00 (0.36)	0.63 (0.37)	0.10 (0.40)	−0.97 (0.43)	**0.002**
Impairment, n (%)	2 (11.8)	0 (0.0)	0 (0.0)	1 (6.7)	1 (7.1)	1 (8.3)	4 (40.0)	
Missing, n	1	0	0	0	0	0	0	
**Affected Hip Flexion, LS mean (SE)**	−3.48 (0.96)	−1.18 (0.81)	−1.55 (0.88)	−1.01 (0.90)	−0.45 (0.89)	−1.28 (0.97)	−0.22 (1.00)	0.322
Impairment, n (%)	6 (54.5)	5 (31.3)	5 (38.5)	4 (30.8)	2 (15.4)	4 (36.4)	2 (20.0)	
Missing, n	7	1	3	2	1	1	0	
**Unaffected Hip Flexion, LS mean (SE)**	−0.38 (0.31)	−0.15 (0.31)	−0.01 (0.32)	−0.07 (0.33)	−0.16 (0.34)	−0.35 (0.36)	−0.47 (0.39)	0.893
Impairment, n (%)	4 (23.5)	3 (17.6)	1 (6.3)	2 (13.3)	1 (7.1)	2 (16.7)	2 (20.0)	
Missing, n	1	0	0	0	0	0	0	
**Right-Sided Tumor**	n = 17	n = 16	n = 15	n = 13	n = 11	n = 4	n = 3	
**Unaffected Ankle Dorsiflexion, LS mean (SE)**	−2.80 (0.27)	−3.04 (0.28)	−3.22 (0.29)	−3.50 (0.31)	−3.46 (0.33)	−3.57 (0.54)	−4.15 (0.62)	0.382
Impairment, n (%)	15 (88.2)	15 (93.8)	13 (92.9)	13 (100.0)	11 (100.0)	4 (100.0)	3 (100.0)	
Missing, n	0	0	1	0	0	0	0	
**Affected Ankle Dorsiflexion, LS mean (SE)**	−3.18 (0.55)	−3.62 (0.53)	−5.34 (0.61)	−4.68 (0.71)	−4.44 (0.68)	−4.60 (1.84)	−3.89 (1.83)	0.057
Impairment, n (%)	13 (76.5)	13 (92.9)	10 (100.0)	8 (100.0)	7 (100.0)	1 (100.0)	1 (100.0)	
Missing, n	4	2	5	5	4	3	2	
**Unaffected Ankle Plantarflexion, LS mean (SE)**	−0.67 (0.34)	−0.96 (0.34)	−0.98 (0.35)	−1.25 (0.39)	−0.86 (0.39)	0.26 (0.58)	−1.40 (0.65)	0.080
Impairment, n (%)	5 (29.4)	3 (18.8)	2 (14.3)	5 (38.5)	3 (27.3)	0 (0.0)	2 (66.7)	
Missing, n	0	0	1	0	0	0	0	
**Affected Ankle Plantarflexion, LS mean (SE)**	−1.07 (0.42)	−1.49 (0.40)	−2.47 (0.44)	−1.92 (0.56)	−1.25 (0.52)	2.14 (1.19)	2.29 (1.19)	**<0.001**
Impairment, n (%)	4 (30.8)	8 (53.3)	5 (50.0)	4 (50.0)	4 (57.1)	0 (0.0)	0 (0.0)	
Missing, n	4	1	5	5	4	3	2	
**Unaffected Knee Flexion, LS mean (SE)**	0.02 (0.38)	0.50 (0.39)	0.35 (0.41)	0.44 (0.44)	−0.60 (0.45)	0.60 (0.70)	−0.51 (0.78)	0.251
Impairment, n (%)	2 (11.8)	1 (6.3)	1 (7.1)	1 (7.7)	3 (27.3)	1 (25.0)	1 (33.3)	
Missing, n	0	0	1	0	0	0	0	
**Affected Knee Flexion, LS mean (SE)**	−5.74 (1.55)	−3.85 (1.59)	−8.29 (1.81)	−7.12 (2.01)	−6.60 (2.05)	−4.74 (3.96)	−3.27 (3.96)	0.373
Impairment, n (%)	10 (66.7)	8 (57.1)	6 (60.0)	7 (77.8)	6 (75.0)	1 (50.0)	1 (50.0)	
Missing, n	2	2	5	4	3	1	1	
**Unaffected Hip Flexion, LS mean (SE)**	−0.67 (0.49)	−0.24 (0.50)	−0.72 (0.51)	0.42 (0.56)	−0.25 (0.57)	−0.24 (0.86)	0.52 (0.96)	0.687
Impairment, n (%)	6 (35.3)	2 (12.5)	3 (21.4)	0 (0.0)	2 (18.2)	0 (0.0)	0 (0.0)	
Missing, n	0	0	1	0	0	0	0	
**Affected Hip Flexion, LS mean (SE)**	−2.98 (0.77)	−1.73 (0.76)	−2.74 (0.83)	−1.79 (0.86)	−1.17 (0.92)	−1.01 (1.42)	−1.08 (1.61)	0.739
Impairment, n (%)	10 (66.7)	7 (46.7)	7 (58.3)	6 (50.0)	5 (50.0)	1 (25.0)	1 (33.3)	
Missing, n	2	1	3	1	1	0	0	

LS = least squared, SE = standard error. Bold indicates statistical significance.

### 3.5. Long-Term Outcomes

The subset of survivors (n = 14) that were enrolled in SJLIFE had a mean age of 19.0 (standard deviation (SD) 3.04) years at SJLIFE evaluation (Table 5). Mean time since last clinical evaluation was 4.68 years (SD 2.04). Overall, physical performance test scores were less than expected compared to the overall SJLIFE population (mean z-score −0.56, SD 1.59) and 27.3% had scores indicating impairment in performance. Survivors did not demonstrate impairment in TUG or BOT testing. Those with left-sided tumors (n = 10) had results below SJLIFE normative scores for bilateral quadriceps strength, bilateral ankle dorsiflexion strength, and bilateral ankle dorsiflexion ROM. Those with right-sided tumors (n = 4) had results below SJLIFE normative scores for unaffected quadriceps strength and bilateral ankle dorsiflexion ROM. Due to amputation or other clinical exclusions, affected quadriceps strength and ankle dorsiflexion strength were not measured. Unaffected ankle dorsiflexion strength was comparable to SJLIFE normative scores (z-score 0.27, SD 1.64). Physical performance test scores were similar by surgery type, but survivors with a tumor located in the ilium or femur had lower scores than survivors with a tumor in the tibia or fibula (Supplemental Table S5). While there was a higher proportion of survivors with a history of limb salvage surgery with strength or ROM impairment at long-term follow-up compared to the proportion of impairment among those with a history of amputation, more survivors with limb-sparing surgery were able to complete these tests.

## 4. Discussion

Major surgical interventions including amputation and limb salvage procedures are associated with poor functional outcomes among survivors of childhood osteosarcoma [21]. Understanding functional trajectories from diagnosis through treatment and into long-term survivorship is important to guide early intervention. In childhood patients and survivors with lower extremity or pelvic osteosarcoma, we found overall trends towards improvement in functional abilities, strength, and ROM from diagnosis to 48-month follow-up; however, improvements were not consistent across time points. Scores on functional assessments declined or were stagnant in the months following surgery and the biggest improvement in scores occurred in the first 18 months after completion of therapy and then remained similar from 18-month to 48-month follow-up. Despite improvements in scores, a large proportion of survivors demonstrated marked impairment throughout the duration of follow-up. Finally, for those evaluated in long-term follow-up, measures of overall function remained below population normative values, as did strength and ROM.

A previous study among survivors of childhood lower extremity and pelvic osteosarcoma that evaluated subjective measurements of function at a mean of 21 years post-diagnosis found that about 70% reported some level of impairment [3]. In the current study, objective measures of function indicated higher levels of impairment throughout the study period. About 90% of participants had functional impairment prior to therapy, prior to surgery, and at the end of therapy. This high proportion of survivors with documented impairment persisted through 6 months post-therapy before decreasing to about 70% at 18 months and increasing back to 80% at 48-month follow-up, respectively. One potential contributor to the higher proportion of impairment observed in our study is the age of participants. With a mean age of 13.1 years at diagnosis, many were skeletally immature at the time of treatment, placing them at risk for postoperative complications due to factors such as ongoing bone development, limb length discrepancy, and inadequate soft tissue storage [22]. We did find improvement in scores on the FMA in the early survivorship period with the largest increases found from the end of therapy to 6-month follow-up and from 6-month follow-up to 18-month follow-up. Our findings are consistent with an earlier longitudinal study that included objective measures of physical function among children and adolescents with malignant bone tumors around the knee joint, showing significant improvements in functional performance tests between 3 and 12 months and 12 and 24 months post-surgery, with the biggest improvements occurring within the first 12 months [9]. This suggests that despite the potential for improvement in functional outcomes among survivors of childhood lower extremity and pelvic osteosarcoma in the early survivorship period, impairment remains common and persistent.

Our results point to the importance of maximizing functional improvement in the early survivorship period to minimize long-term impairment. Additionally, given the large proportion of impairment seen prior to treatment initiation, prehabilitation should be considered in this population. Prehabilitation in the 10–12 weeks prior to surgery has been found to be feasible among children and adolescents with lower extremity sarcoma [23]. In an intervention including three 60 min physical therapy sessions per week consisting of endurance, strengthening, and stretching exercises while undergoing neoadjuvant chemotherapy prior to limb-sparing surgery, the majority of enrolled participants completed ≥50% of the planned sessions and those in the intervention group scored higher on the FMA at 10–12 weeks post-surgery compared with those who received usual care [23]. Rehabilitation during adjuvant therapy among pediatric patients with lower extremity sarcoma has also been found to be feasible and beneficial [24]. An intervention including strength, coordination, flexibility, and endurance exercises delivered during inpatient therapy was associated with increased physical activity levels at up to 18 months of follow-up among participants compared to controls [24]. Finally, as we saw an increase in the proportion of survivors with impairment from the 18- to 48-month follow-up time points, interventions designed to maintain and/or incrementally improve physical function may be important to mitigate functional decline in long-term survivors of childhood lower extremity osteosarcoma. In a randomized controlled trial including adolescents and young adults with lower extremity sarcoma with a history of limb-sparing surgery at a mean of 5.5 years post-surgery, participation in a supervised remote 8-week intervention including strength, balance, coordination, and mobility exercises was associated with improvements in gait and function [25].

Similarly to scores on the FMA, we found improvements in both ipsilateral and contralateral quadriceps strength from 6-month to 48-month follow-up; however, impairment in ipsilateral strength persisted throughout follow-up. For those with left-sided tumors, while there were improvements in ipsilateral quadriceps strength, about 25% of survivors were impaired at both 6- and 18-month follow-up. Fewer survivors had right-sided tumors, and similarly, while scores improved throughout follow-up, one-third had ipsilateral quadriceps strength impairment at both 6- and 48-month follow-up. These results are similar to the functional findings in our study, highlighting that improvements in strength can occur among survivors, though impairment remains evident. Strength-focused interventions have shown promise among survivors of childhood lower extremity sarcoma. Survivors who participated in a supervised, progressive, 6-week strength training intervention had improvements in ipsilateral strength, and importantly improvements in functional and quality of life outcomes as well [26]. Range of motion has also been shown to correlate with function and quality of life in survivors of childhood lower extremity sarcoma [27], and our data demonstrate that ROM scores in multiple joints, both ipsilateral and contralateral to tumor location, are low throughout treatment and follow-up. Exercises targeted towards maintaining and improving both ipsilateral and contralateral ROM should be incorporated in the care of survivors of childhood lower extremity osteosarcoma, not only in the post-surgical period, but throughout long-term follow-up.

While interventions aimed at improving function and strength have demonstrated success at specific time points across the cancer continuum among childhood lower extremity sarcoma patients and survivors, it is not clear if these improvements are durable. Among the subset of survivors that were evaluated in long-term follow-up, impairments remained evident. About one-third of those evaluated had marked impairment on the PPT, a comprehensive measure of function. Among those with left-sided tumors, ipsilateral quadriceps and ankle dorsiflexion strength scores were below population means and scores reached the impairment threshold in 20% of those assessed. Among the few survivors evaluated in long-term follow-up with right-sided tumors, ipsilateral strength was unable to be assessed due to participant limitations. Ipsilateral ankle dorsiflexion ROM was impaired in about half of participants who underwent evaluation. These findings underscore the need for longitudinal studies to evaluate whether the functional, strength, and ROM gains resulting from interventions during the treatment and early survivorship periods are sustained longer-term. Additionally, studies have shown that survivors of childhood cancer experience accelerated functional and strength declines as they age [28], thus it may be particularly important for survivors of childhood lower extremity sarcoma to participate in targeted training aimed at mitigating these declines.

An important factor when considering the results of this study is the use of bevacizumab, a monoclonal antibody directed against the vascular endothelial growth factor. Bevacizumab is known to be associated with increased infection risk in cancer patients [29], and in this trial was associated with a high rate (52%) of wound complications, a risk factor for infection, after surgery [11]. Wound complications required additional surgical intervention including debridement and deep implant removal and were associated with increased time to therapy completion. In a retrospective review from our institution, pediatric and young adult patients with bone malignancies who developed wound infections were more likely to develop orthopedic device infections and less likely to have good functional outcomes [30]. Thus, the use of bevacizumab may have contributed to poor functional outcomes in this study, and, when using antiangiogenic agents for the treatment of lower extremity sarcoma, wound complications and the associated risk of poor functional outcomes should be considered and closely monitored.

There are several limitations to consider when interpreting the results of our study. Due to clinical status or other limiting factors, participants were lost at each study time point. This left a small sample size, particularly for 18-month, 48-month, and long-term follow-up. As participants with relapse or progression were likely not included in later assessment time points, better function may have been observed in the remaining participants than would have been seen if outcomes in all original patients were able to be measured. This also limited our ability to add covariates to the statistical model to determine how confounding factors such as skeletal maturation, level of surgery, and postoperative recovery time impacted outcomes. While difficult due to the overall rarity of childhood lower extremity and pelvic osteosarcoma and the aggressive nature of the disease and its treatment, larger studies evaluating longitudinal functional and strength outcomes are needed in this population. Additionally, referral to physical and occupational therapies are standard following surgical treatment of osteosarcoma [31], but we were unable to assess the association of participation in these interventions with functional and strength outcomes. Finally, the use of different functional measures in long-term SJLIFE follow-up limits the ability to compare outcomes to earlier time points, though percent impaired remains an important comparison.

## 5. Conclusions

We demonstrated that survivors of childhood lower extremity and pelvic osteosarcoma have a high burden of functional, strength, and ROM impairment that persists from diagnosis to long-term follow-up. However, scores on objective measures do show improvement, particularly within the first 18 months post-therapy. These data suggest windows during which interventions aimed at improving function and strength may be particularly beneficial, but, given the persistent impairment found in this study, suggest that a lifelong focus on mitigating functional, strength, and ROM decline is needed in survivors.

## Figures and Tables

**Table 5:** Age- and sex-specific z-scores (mean and SD) and percent of impairment (below −1.5 SDs) for functional and strength measures in those who participated in SJLIFE (n = 14).

All Participants	
**Age at long-term follow-up, mean (SD)**	19.00 (3.04)
**Time since last clinical evaluation, mean (SD)**	4.68 (2.04)
**Sex**	
Male, n (%)	7 (50.0)
**PPT z-score, mean (SD)**	−0.56 (1.59)
Impairment, n (%)	3 (27.3)
**TUG time z-score, mean (SD)**	1.52 (1.11)
Impairment, n (%)	0 (0.0)
**BOT z-score, mean (SD)**	2.86 (0.38)
Impairment, n (%)	0 (0.0)
**Grip strength, mean (SD)**	0.39 (1.23)
Impairment, n (%)	1 (7.1)
**Left side tumor location** n = 10	
**Affected quadriceps strength z-score, mean (SD)**	−1.62 (1.31)
Impairment, n (%)	2 (20.0)
Amputation, n (%)	1 (10.0)
Other, n (%)	6 (60.0)
**Unaffected quadriceps strength z-score, mean (SD)**	−0.91 (0.89)
Impairment, n (%)	1 (10.0)
Amputation, n (%)	0 (0.0)
Other, n (%)	1 (10.0)
**Affected ankle dorsiflexion strength z-score, mean (SD)**	−1.13 (0.59)
Impairment, n (%)	2 (20.0)
Amputation, n (%)	1 (10.0)
Other, n (%)	4 (40.0)
**Unaffected ankle dorsiflexion strength z-score, mean (SD)**	−0.16 (0.99)
Impairment, n (%)	0 (0.0)
Amputation, n (%)	0 (0.0)
Other, n (%)	1 (10.0)
**Affected ankle dorsiflexion ROM z-score, mean (SD)**	−1.03 (1.80)
Impairment, n (%)	4 (40.0)
Amputation, n (%)	1 (10.0)
**Unaffected ankle dorsiflexion ROM z-score, mean (SD)**	−0.01 (1.34)
Impairment, n (%)	1 (10.0)
Amputation, n (%)	0 (0.0)
**Right side tumor location** n = 4	
**Unaffected quadriceps strength z-score, mean (SD)**	−0.53 (0.83)
Impairment, n (%)	1 (75.0)
Amputation, n (%)	0 (0.0)
Other, n (%)	0 (0.0)
**Affected quadriceps strength z-score, mean (SD)**	N/A
Impairment, n (%)	0 (0.0)
Amputation, n (%)	2 (50.0)
Other, n (%)	2 (50.0)
**Unaffected ankle dorsiflexion strength z-score, mean (SD)**	0.27 (1.64)
Impairment, n (%)	0 (0.0)
Amputation, n (%)	0 (0.0)
Other, n (%)	0 (0.0)
**Affected ankle dorsiflexion strength z-score, mean (SD)**	N/A
Impairment, n (%)	0 (0.0)
Amputation, n (%)	2 (50.0)
Other, n (%)	2 (50.0)
**Unaffected ankle dorsiflexion ROM z-score, mean (SD)**	−0.05 (0.74)
Impairment, n (%)	0 (0.0)
Amputation, n (%)	0 (0.0)
**Affected ankle dorsiflexion ROM z-score, mean (SD)**	−2.80 (1.77)
Impairment, n (%)	2 (50.0)
Amputation, n (%)	2 (50.0)

SD = standard deviation; PPT = physical performance test; TUG = timed up and go; BOT = Bruininks–Oseretsky test of motor proficiency; N/A = mean not evaluable due to missing data.

## Data Availability

These data will be made available via Zenodo.

## Supplementary Materials

The following supporting information can be downloaded at: https://www.mdpi.com/article/10.3390/cancers18111790/s1, Table S1: OS08 Treatment Regimen, Table S2: Mean (SE) and percent impairment of total functional mobility assessment score based on age and sex median normative scores by surgery type and tumor location, Table S3: Age- and sex-specific z-scores (least squared mean and SE) and percent of impairment (below −1.5 SEs) for isometric knee extension and ankle dorsiflexion strength measures by surgery type and tumor location, Table S4: Age- and sex-specific z-scores (least squared mean and SE) and percent of impairment (below −1.5 SEs) for knee and ankle active range of motion by surgery type and tumor location, Table S5: Age- and sex-specific z-scores (mean and SD) and percent of impairment (below −1.5 SDs) for functional and strength measures in those who participated in the SJLIFE cohort by surgery type and tumor location.
