# Supplementary material for: Longitudinal Functional Outcomes Among Survivors of Childhood Lower Extremity Osteosarcoma
Source: Cancers (Basel). 2026 May 29;18(11):1790. doi: 10.3390/cancers18111790 (PMC13256059; doi:10.3390/cancers18111790)
Supplement: Supplementary file 1 [file cancers-18-01790-s001.zip › cancers-4269243-supplementary.pdf]

Supplemental Table S1. OS08 Treatment Regimen

Drug	Treatment Strata
Cisplatin	All
Doxorubicin	All
Methotrexate	All
Bevacizumab	All
Ifosfamide	Those with localized unresectable primary tumors or metastatic disease
Etoposide	Those with localized unresectable primary tumors or metastatic disease

St. Jude - Confidential

Supplemental Table S2. Mean (SE) and percent impairment of total functional mobility assessment score based on age and sex median normative scores by surgery type and tumor location

	Pre-Therapy	Week 10	Week 20-22	End of Therapy	6 Month	18 Month	48 Month	p-trend
**Surgery Type: Amputation**								
**FMA score, LS mean (SE)**	25.3 (5.1)	22.7 (4.7)	27.0 (5.4)	29.0 (5.1)	35.6 (5.5)	47.2 (6.1)	50.2 (6.8)	**0.027**
Impaired, n (%)	6 (100.0)	7 (100.0)	5 (100.0)	6 (100.0)	5 (100.0)	3 (75.0)	1 (33.3)	
Not impaired, n (%)	0 (0.0)	0 (0.0)	0 (0.0)	0 (0.0)	0 (0.0)	1 (25.0)	2 (66.7)	
Not completed	2	1	2	1	0	0	0	
**Surgery Type: Limb Salvage**								
**FMA score, LS mean (SE)**	27.2 (2.6)	32.8 (2.7)	30.2 (2.9)	37.2 (2.8)	42.4 (2.9)	47.9 (3.4)	53.0 (3.5)	**<0.0001**
Impaired, n (%)	21 (84.0)	22 (91.7)	18 (100.0)	19 (90.5)	17 (85.0)	8 (66.7)	7 (70.0)	
Not impaired, n (%)	4 (16.0)	2 (8.3)	0 (0.0)	2 (9.5)	3 (15.0)	4 (33.3)	3 (30.0)	
Not completed	2	1	8	0	0	0	0	
**Tumor Location: Ilium or Femur (Upper)**								
**FMA score, LS mean (SE)**	32.6 (3.9)	30.3 (3.8)	29.9 (4.2)	34.5 (4.3)	42.0 (4.5)	47.1 (5.1)	54.5 (5.3)	**0.007**
Impaired, n (%)	18 (90.0)	18 (94.7)	14 (100.0)	17 (94.4)	16 (94.1)	7 (70.0)	7 (87.5)	
Not impaired, n (%)	2 (10.0)	1 (5.3)	0 (0.0)	1 (5.6)	1 (5.9)	3 (30.0)	1 (12.5)	
Not completed	2	2	5	0	0	0	0	
**Tumor Location: Tibia or Fibula (Lower)**								
**FMA score, LS mean (SE)**	23.6 (3.0)	30.5 (3.0)	29.2 (3.2)	35.8 (3.1)	40.3 (3.2)	48.0 (3.7)	50.7 (3.9)	**<0.0001**
Impaired, n (%)	9 (81.8)	11 (91.7)	9 (100.0)	8 (88.9)	6 (75.0)	4 (66.7)	2 (20.0)	
Not impaired, n (%)	2 (18.2)	1 (9.3)	0 (0.0)	1 (11.1)	2 (25.0)	2 (33.3)	5 (80.0)	
Not completed	2	0	3	1	0	0	0	

FMA = functional mobility assessment; LS = least squared; SE = standard error

Supplemental Table S3. Age- and sex-specific z-scores (least squared mean and SE) and percent of impairment (below -1.5 SEs) for isometric knee extension and ankle dorsiflexion strength measures by surgery type and tumor location

	6 Month	18 Month	48 Month	p-trend
**Surgery Type: Amputation**	n=5	n=4	n=3	
**Left Quadriceps, LS mean (SE)**	2.03 (1.40)	1.18 (1.40)	1.94 (1.40)	0.060
Impaired, n (%)	0 (0.0)	0 (0.0)	0 (0.0)	
Missing, n	2	1	1	
**Right Quadriceps, LS mean (SE)**	1.25 (2.24)	3.45 (2.24)	4.28 (2.43)	0.385
Impaired, n (%)	0 (0)	0 (0.0)	0 (0.0)	
Missing, n	3	2	2	
**Left Ankle Dorsiflexion, LS mean (SE)**	0.46 (1.12)	-0.38 (1.12)	1.34 (1.21)	0.199
Impaired, n (%)	1 (33.3)	0 (0.0)	0 (0.0)	
Missing, n	2	1	1	
**Right Ankle Dorsiflexion, LS mean (SE)**	0.88 (1.74)	6.03 (2.46)	5.75 (2.46)	[a]
Impaired, n (%)	0 (0.0)	0 (0.0)	0 (0.0)	
Missing, n	3	3	2	
**Surgery Type: Limb Salvage**	n=20	n=12	n=10	
**Left Quadriceps, LS mean (SE)**	0.49 (0.48)	0.41 (0.53)	1.33 (0.56)	0.061
Impaired, n (%)	3 (16.7)	1 (11.1)	2 (20.0)	
Missing, n	2	3	0	
**Right Quadriceps, LS mean (SE)**	1.96 (0.63)	3.11 (0.71)	3.83 (0.78)	0.066
Impaired, n (%)	2 (11.1)	0 (0.0)	0 (0.0)	
Missing, n	2	0	0	
**Left Ankle Dorsiflexion, LS mean (SE)**	0.25 (0.50)	0.87 (0.57)	1.40 (0.62)	0.203
Impaired, n (%)	4 (22.2)	3 (27.3)	1 (10.0)	
Missing, n	2	1	0	
**Right Ankle Dorsiflexion, LS mean (SE)**	1.16 (0.54)	1.99 (0.62)	1.42 (0.72)	0.249
Impaired, n (%)	4 (20.0)	0 (0.0)	1 (11.1)	
Missing, n	0	0	1	
**Tumor Location: Ilium or Femur (Upper)**	n=17	n=10	n=8	
**Left Quadriceps, LS mean (SE)**	0.96 (0.58)	1.04 (0.62)	1.88 (0.66)	0.091
Impaired, n (%)	1 (6.7)	0 (0.0)	1 (12.5)	
Missing, n	2	2	0	
**Right Quadriceps, LS mean (SE)**	1.86 (0.65)	2.73 (0.73)	3.43 (0.83)	0.221
Impaired, n (%)	1 (7.7)	0 (0.0)	0 (0.0)	
Missing, n	4	1	1	
**Left Ankle Dorsiflexion, LS mean (SE)**	0.85 (0.46)	1.90 (0.55)	2.34 (0.60)	0.082
Impaired, n (%)	2 (13.3)	0 (0.0)	0 (0.0)	
Missing, n	2	1	0	
**Right Ankle Dorsiflexion, LS mean (SE)**	1.34 (0.49)	1.70 (0.58)	1.12 (0.71)	0.623
Impaired, n (%)	2 (13.3)	0 (0.0)	1 (16.7)	
Missing, n	2	1	2	
**Tumor Location: Tibia or Fibula (Lower)**	n=8	n=6	n=5	
**Left Quadriceps, LS mean (SE)**	0.32 (0.78)	-0.40 (0.82)	0.83 (0.86)	0.152
Impaired, n (%)	2 (33.3)	1 (25.0)	1 (25.0)	
Missing, n	2	2	1	
**Right Quadriceps, LS mean (SE)**	1.96 (1.18)	3.94 (1.27)	4.67 (1.40)	0.136

St. Jude - Confidential

Impaired, n (%)	1 (14.3)	0 (0.0)	0(0.0)	
Missing, n	1	1	1	
**Left Ankle Dorsiflexion, LS mean (SE)**	-1.03 (0.59)	-1.75 (0.63)	-0.27 (0.70)	0.110
Impaired, n (%)	3 (50.0)	3 (60.0)	1 (25.0)	
Missing, n	2	1	1	
**Right Ankle Dorsiflexion, LS mean (SE)**	0.70 (1.19)	3.25 (1.36)	2.64 (1.45)	0.120
Impaired, n (%)	2 (28.6)	0 (0.0)	0 (0.0)	
Missing, n	1	2	1	

LS = least squared, SE = standard error

[a]p-value could not be estimated

St. Jude - Confidential

Table S4. Age- and sex-specific z-scores (least squared mean and SE) and percent of impairment (below -1.5 SEs) for knee and ankle active range of motion by surgery type and tumor location

	Pre-Therapy	Week 10	Week 20-22	End of Therapy	6 Month	18 Month	48 Month	p-trend
**Surgery Type: Amputation**	n=8	n=8	n=7	n=7	n=5	n=4	n=3	
**Left Ankle Dorsiflexion, LS mean (SE)**	-2.91 (0.53)	-2.96 (0.53)	-3.71 (0.56)	-3.97 (0.57)	-3.91 (0.62)	-3.98 (0.70)	-4.08 (0.79)	0.530
Impairment, n (%)	5 (83.3)	6 (100.0)	5 (100.0)	5 (100.0)	4 (100.0)	3 (100.0)	2 (100.0)	
Missing, n	2	2	2	2	1	1	1	
**Right Ankle Dorsiflexion, LS mean (SE)**	-3.06 (0.61)	-3.00 (0.56)	-3.16 (0.74)	-3.39 (0.90)	-1.80 (0.93)	-3.38 (1.26)	-2.74 (1.23)	0.907
Impairment, n (%)	4 (80.0)	5 (83.3)	3 (100.0)	2 (100.0)	1 (100.0)	1 (100.0)	1 (100.0)	
Missing, n	3	2	4	5	4	3	2	
**Left Ankle Plantarflexion, LS mean (SE)**	-0.83 (0.46)	-0.65 (0.56)	-0.29 (0.48)	-1.79 (0.49)	-0.43 (0.52)	-0.24 (0.57)	-2.18 (0.61)	**0.001**
Impairment, n (%)	2 (33.3)	0 (0.0)	0 (0.0)	3 (60.0)	1 (25.0)	0 (0.0)	2 (10.0)	
Missing, n	2	2	2	2	1	1	1	
**Right Ankle Plantarflexion, LS mean (SE)**	-1.01 (0.80)	-1.69 (0.67)	-2.93 (1.02)	-0.50 (1.25)	0.49 (1.76)	-0.81 (1.78)	0.64 (1.75)	0.520
Impairment, n (%)	2 (40.0)	4 (57.1)	1 (33.3)	0 (0.0)	0 (0.0)	0 (0.0)	0 (0.0)	
Missing, n	3	1	4	5	4	3	2	
**Left Knee Flexion, LS mean (SE)**	0.42 (0.67)	0.82 (0.67)	0.29 (0.71)	0.15 (0.77)	-0.43 (0.78)	-0.47 (0.86)	-0.50 (0.95)	0.860
Impairment, n (%)	0 (0.0)	0 (0.0)	0 (0.0)	1 (20.0)	1 (20.0)	1 (25.0)	1 (33.3)	
Missing, n	1	1	1	2	0	0	0	
**Right Knee Flexion, LS mean (SE)**	-1.56 (1.64)	-1.27 (1.68)	-1.26 (1.71)	-0.06 (1.73)	-2.97 (1.77)	-3.03 (1.77)	-2.89 (1.73)	**0.018**
Impairment, n (%)	2 (28.6)	1 (16.7)	0 (0.0)	0 (0.0)	0 (0.0)	0 (0.0)	0 (0.0)	
Missing, n	1	2	4	4	3	2	1	
**Left Hip Flexion, LS mean (SE)**	-0.44 (0.68)	0.45 (0.68)	0.14 (0.73)	0.35 (0.73)	0.16 (0.80)	-0.27 (0.88)	0.26 (0.98)	0.959
Impairment, n (%)	3 (42.9)	0 (0.0)	0 (0.0)	0 (0.0)	1 (20.0)	1 (25.0)	0 (0.0)	
Missing, n	1	1	1	1	0	0	0	
**Right Hip Flexion, LS mean (SE)**	-1.90 (0.82)	-1.25 (0.81)	-1.20 (0.86)	-0.60 (0.89)	-0.46 (1.03)	-1.90 (1.05)	-2.60 (1.12)	0.728
Impairment, n (%)	4 (57.1)	3 (42.9)	2 (33.3)	2 (33.3)	1 (25.0)	2 (50.0)	1 (33.3)	
Missing, n	1	1	1	1	1	0	0	

**Surgery Type: Limb Salvage**	n=27	n=25	n=24	n=21	n=20	n=12	n=10	
**Left Ankle Dorsiflexion, LS mean (SE)**	-3.18 (0.29)	-3.22 (0.30)	-3.80 (0.30)	-4.23 (0.31)	-3.61 (0.32)	-3.36 (0.39)	-3.39 (0.39)	**0.044**
Impairment, n (%)	24 (92.3)	21 (91.3)	19 (86.4)	21 (100.0)	20 (100.0)	9 (81.8)	10 (100.0)	
Missing, n	1	2	2	0	0	1	0	
**Right Ankle Dorsiflexion, LS mean (SE)**	-3.39 (0.31)	-3.97 (0.31)	-3.87 (0.32)	-4.01 (0.33)	-3.82 (0.38)	-3.76 (0.39)	-2.80 (0.31)	**0.009**
Impairment, n (%)	22 (88.0)	22 (88.0)	22 (95.7)	21 (100.0)	19 (95.0)	12 (100.0)	10 (100.0)	
Missing, n	2	0	1	0	0	0	0	
**Left Ankle Plantarflexion, LS mean (SE)**	-1.38 (0.32)	-1.47 (0.34)	-1.86 (0.34)	-1.14 (0.35)	-1.20 (0.36)	-1.31 (0.44)	-1.38 (0.32)	**0.024**
Impairment, n (%)	13 (50.0)	12 (52.2)	11 (50.0)	7 (33.3)	8 (40.0)	6 (45.5)	2 (20.0)	
Missing, n	1	2	2	0	0	1	0	0.135
**Right Ankle Plantarflexion, LS mean (SE)**	-1.03 (0.27)	-1.43 (0.27)	-1.65 (0.27)	-1.10 (0.29)	-1.14 (0.29)	-0.90 (0.36)	-0.27 90.38)	
Impairment, n (%)	11 (44.0)	12 (48.0)	12 (52.2)	10 (47.6)	9 (45.0)	3 (25.0)	2 (20.0)	
Missing, n	2	0	1	0	0	0	0	
**Left Knee Flexion, LS mean (SE)**	-3.61 (1.10)	-0.98 (1.06)	-4.79 (1.08)	-2.98 (1.11)	-2.09 (1.15)	-2.63 (1.37)	-3.63 (1.41)	**0.007**
Impairment, n (%)	9 (42.9)	5 (21.7)	10 (47.6)	9 (45.0)	10 (52.6)	7 (63.3)	6 (60.0)	
Missing, n	6	2	3	1	1	1	0	
**Right Knee Flexion, LS mean (SE)**	-2.58 (1.06)	-1.24 (1.06)	-3.41 (1.08)	-3.00 (1.12)	-2.95 (1.15)	-2.87 (1.32)	-3.66 (1.36)	0.294
Impairment, n (%)	10 (40.0)	7 (28.0)	6 (26.1)	8 (38.1)	7 (35.0)	2 (16.7)	5 (50.0)	
Missing, n	2	0	1	0	0	0	0	
**Left Hip Flexion, LS mean (SE)**	-2.19 (0.65)	-0.90 (0.60)	-1.49 (0.64)	-0.56 (0.66)	-0.60 (0.68)	-1.10 (0.85)	0.07 (0.89)	0.197
Impairment, n (%)	9 (42.9)	7 (28.0)	8 (38.1)	4 (20.0)	3 (15.8)	3 (27.3)	2 (20.0)	
Missing, n	6	0	3	1	1	1	0	
**Right Hip Flexion, LS mean (SE)**	-1.47 (0.51)	-0.81 (0.52)	-1.09 (0.53)	-1.27 (0.54)	-0.99 (0.55)	-1.01 (0.61)	-1.28 (0.51)	0.892
Impairment, n (%)	10 (40.0)	7 (28.0)	6 (27.3)	6 (28.6)	5 (25.0)	1 (8.3)	2 (20.0)	
Missing, n	2	0	2	0	0	0	0	
**Tumor Location: Ilium or Femur (Upper)**	n=22	n=21	n=19	n=18	n=17	n=10	n=8	
**Left Ankle Dorsiflexion, LS mean (SE)**	-2.92 (0.23)	-3.10 (0.25)	-3.49 (0.25)	-3.71 (0.25)	-3.29 (0.25)	-2.96 (0.32)	-3.01 (0.34)	0.255
Impairment, n (%)	19 (95.0)	17 (94.4)	15 (88.2)	17 (100.0)	17 (100.0)	7 (77.8)	8 (100.0)	

Missing, n	2	3	2	0	0	1	0	
**Right Ankle Dorsiflexion, LS mean (SE)**	-2.42 (0.30)	-3.26 (0.29)	-3.27 (0.31)	-3.40 (0.31)	-3.38 (0.32)	-3.51 (0.39)	-3.62 (0.40)	0.063
Impairment, n (%)	15 (83.3)	16 (84.2)	15 (93.8)	16 (100.0)	14 (93.3)	9 (100.0)	7 (100.0)	
Missing, n	4	2	3	2	2	1	1	
**Left Ankle Plantarflexion, LS mean (SE)**	-1.73 (0.32)	-1.07 (0.33)	-1.41 (0.34)	-1.10 (0.34)	-0.99 (0.35)	-0.77 (0.46)	-0.29 (0.48)	0.153
Impairment, n (%)	11 (55.0)	6 (33.3)	6 (35.3)	6 (35.3)	6 (35.3)	3 (33.3)	2 (25.0)	
Missing, n	2	3	2	1	0	1	0	
**Right Ankle Plantarflexion, LS mean (SE)**	-1.24 (0.27)	-1.39 (0.26)	-1.37 (0.28)	-1.00 (0.29)	-1.06 (0.30)	-0.67 (0.37)	0.12 (0.41)	0.067
Impairment, n (%)	8 (44.4)	9 (45.0)	6 (37.5)	7 (43.8)	6 (40.0)	2 (22.2)	1 (14.3)	
Missing, n	4	1	3	2	2	1	1	
**Left Knee Flexion, LS mean (SE)**	-3.79 (1.26)	-1.30 (1.19)	-4.01 (1.24)	-2.44 (1.27)	-1.74 (1.29)	-1.42 (1.51)	-3.02 (1.57)	0.103
Impairment, n (%)	5 (31.3)	4 (21.1)	8 (50.0)	7 (43.8)	8 (50.0)	5 (55.6)	4 (50.0)	
Missing, n	6	2	3	2	1	1	0	
**Right Knee Flexion, LS mean (SE)**	-3.44 (1.18)	-1.72 (1.28)	-2.62 (1.22)	-2.96 (1.23)	-2.20 (1.25)	-3.04 (1.37)	-4.24 (1.41)	0.501
Impairment, n (%)	9 (47.4)	7 (35.0)	3 (18.8)	6 (37.5)	6 (40.0)	2 (22.2)	4 (57.1)	
Missing, n	3	1	3	2	2	1	1	
**Left Hip Flexion, LS mean (SE)**	-2.69 (0.68)	-0.94 (0.65)	-0.60 (0.69)	-0.13 (0.70)	0.22 (0.71)	0.27 (0.84)	0.29 (0.68)	**0.025**
Impairment, n (%)	8 (47.1)	5 (25.0)	4 (25.0)	3 (18.8)	2 (12.5)	1 (11.1)	2 (25.0)	
Missing, n	5	1	3	2	1	1	0	
**Right Hip Flexion, LS mean (SE)**	-2.24 (0.61)	-0.97 (0.61)	-0.54 (0.63)	-1.03 (0.63)	-1.15 (0.64)	-1.31 (0.70)	-2.08 (0.72)	0.337
Impairment, n (%)	10 (52.6)	6 (30.0)	2 (12.5)	4 (23.5)	5 (31.3)	2 (20.0)	3 (37.5)	
Missing, n	3	1	3	1	1	0	0	
**Tumor Location: Tibia or Fibula (Lower)**	n=13	n=12	n=12	n=10	n=8	n=6	n=5	
**Left Ankle Dorsiflexion, LS mean (SE)**	-3.50 (0.54)	-3.28 (0.55)	-4.27 (0.57)	-5.07 (0.59)	-4.36 (0.66)	-4.48 (0.74)	-4.49 (0.76)	0.128
Impairment, n (%)	10 (83.3)	10 (90.9)	9 (90.0)	9 (100.0)	7 (100.0)	5 (100.0)	4 (100.0)	
Missing, n	1	1	2	1	1	1	1	
**Right Ankle Dorsiflexion, LS mean (SE)**	-3.44 (0.51)	-3.35 (0.50)	-5.00 (0.53)	-4.51 (0.61)	-4.44 (0.63)	-3.76 (0.77)	-4.01 (0.75)	0.073
Impairment, n (%)	11 (91.7)	11 (91.7)	0 (100.0)	7 (100.0)	6 (100.0)	4 (100.0)	4 (100.0)	

Missing, n	1	0	2	3	2	2	1	
**Left Ankle Plantarflexion, LS mean (SE)**	-0.54 (0.51)	-1.69 (0.52)	-1.81 (0.53)	-1.66 (0.56)	-1.41 (0.61)	-2.05 (0.69)	-1.28 (0.71)	0.413
Impairment, n (%)	4 (33.3)	6 (54.5)	5 (50.0)	4 (44.4)	3 (42.9)	3 (60.0)	2 (50.0)	
Missing, n	1	1	2	1	1	1	1	
**Right Ankle Plantarflexion, LS mean (SE)**	-0.77 (0.50)	-1.63 (0.49)	-2.53 (0.52)	-1.42 (0.61)	-1.36 (0.66)	-1.89 (0.77)	-1.13 (0.73)	0.145
Impairment, n (%)	5 (41.7)	7 (58.3)	7 (70.0)	3 (42.9)	3 (50.0)	1 (25.0)	1 (25.0)	
Missing, n	1	0	2	3	2	2	1	
**Left Knee Flexion, LS mean (SE)**	-1.18 (1.21)	0.53 (1.24)	-3.00 (1.23)	-2.01 (1.31)	-1.66 (1.40)	-3.07 (1.55)	-2.49 (1.59)	0.086
Impairment, n (%)	4 (33.3)	1 (9.1)	2 (18.2)	3 (33.3)	3 (37.5)	3 (50.0)	3 (60.0)	
Missing, n	1	1	1	1	0	0	0	
**Right Knee Flexion, LS mean (SE)**	-0.76 (1.37)	-0.09 (1.47)	-4.10 (1.53)	-1.82 (1.69)	-3.92 (1.81)	-2.15 (2.06)	-2.03 (1.99)	0.146
Impairment, n (%)	3 (23.1)	1 (9.1)	3 (30.0)	2 (25.0)	1 (14.3)	0 (0.0)	1 (20.0)	
Missing, n	0	1	2	2	1	1	0	
**Left Hip Flexion, LS mean (SE)**	-0.49 (0.85)	0.02 (0.81)	-1.80 (0.83)	-0.44 (0.88)	-1.37 (0.98)	-2.25 (1.12)	-0.50 (1.19)	0.187
Impairment, n (%)	4 (36.4)	2 (16.7)	4 (36.4)	1 (10.0)	2 (25.0)	3 (50.0)	0 (0.0)	
Missing, n	2	0	1	0	0	0	0	
**Right Hip Flexion, LS mean (SE)**	-0.53 (0.53)	-0.73 (0.54)	-1.99 (0.54)	-1.31 (0.59)	-0.33 (0.65)	-1.05 (0.73)	-0.70 (0.76)	0.175
Impairment, n (%)	4 (30.8)	4 (33.3)	6 (50.0)	4 (40.0)	1 (12.5)	1 (16.7)	0 (0.0)	
Missing, n	0	0	0	0	0	0	0	

LS = least squared, SE = standard error

Table S5. Age- and sex-specific z-scores (mean and SD) and percent of impairment (below -1.5 SDs) for functional and strength measures in those who participated in the SJLIFE cohort by surgery type and tumor location

**Surgery Type: Amputation**	
**PPT z-score, mean (SD)**	-0.56 (1.90)
Impairment, n (%)	1 (33.3)
**TUG time z-score, mean (SD)**	0.71 (1.94)
Impairment, n (%)	0 (0.0)
**BOT z-score, mean (SD)**	N/A
Impairment, n (%)	
**Grip strength, mean (SD)**	0.56 (1.30)
Impairment, n (%)	0 (0.0)
**Left Quadriceps strength z-score, mean (SD)**	-0.41 (0.48)
Impairment, n (%)	0 (0.0)
**Right Quadriceps strength z-score, mean (SD)**	-1.28 (N/A)
Impairment, n (%)	0 (0.0)
**Left Ankle Dorsiflexion strength z-score, mean (SD)**	-0.05 (0.26)
Impairment, n (%)	0 (0.0)
**Right Ankle Dorsiflexion strength z-score, mean (SD)**	0.01 (N/A)
Impairment, n (%)	0 (0.0)
**Left Ankle Dorsiflexion ROM z-score, mean (SD)**	-0.67 (0.17)
Impairment, n (%)	0 (0.0)
**Right Ankle Dorsiflexion ROM z-score, mean (SD)**	-0.24 (N/A)
Impairment, n (%)	0 (0.0)
**Surgery Type: Limb Salvage**	
**PPT z-score, mean (SD)**	-0.57 (1.61)
Impairment, n (%)	2 (33.3)
**TUG time z-score, mean (SD)**	1.75 (0.76)
Impairment, n (%)	0 (0.0)

**BOT z-score, mean (SD)**	2.87 (0.38)
Impairment, n (%)	0 (0.0)
**Grip strength, mean (SD)**	0.34 (1.28)
Impairment, n (%)	1 (9.1)
**Left Quadriceps strength z-score, mean (SD)**	-1.23 (1.26)
Impairment, n (%)	3 (60.0)
**Right Quadriceps strength z-score, mean (SD)**	-0.97 (0.94)
Impairment, n (%)	1 (12.5)
**Left Ankle Dorsiflexion strength z-score, mean (SD)**	-0.63 (1.49)
Impairment, n (%)	2 (28.6)
**Right Ankle Dorsiflexion strength z-score, mean (SD)**	-0.18 (1.05)
Impairment, n (%)	0 (0.0)
**Left Ankle Dorsiflexion ROM z-score, mean (SD)**	-0.74 (1.74)
Impairment, n (%)	4 (36.4)
**Right Ankle Dorsiflexion ROM z-score, mean (SD)**	-0.50 (1.80)
Impairment, n (%)	3 (27.3)
**Tumor Location: Ilium or Femur (Upper)**	
**PPT z-score, mean (SD)**	-1.49 (1.67)
Impairment, n (%)	3 (50.0)
**TUG time z-score, mean (SD)**	2.06 (0.70)
Impairment, n (%)	0 (0.0)
**BOT z-score, mean (SD)**	2.87 (0.38)
Impairment, n (%)	0 (0.0)
**Grip strength, mean (SD)**	0.45 (1.45)
Impairment, n (%)	1 (11.1)
**Left Quadriceps strength z-score, mean (SD)**	-1.27 (1.36)
Impairment, n (%)	1 (50.0)
**Right Quadriceps strength z-score, mean (SD)**	-1.24 (0.60)

St. Jude - Confidential

Impairment, n (%)	1 (16.7)
**Left Ankle Dorsiflexion strength z-score, mean (SD)**	-0.34 (1.60)
Impairment, n (%)	2 (33.3)
**Right Ankle Dorsiflexion strength z-score, mean (SD)**	-0.49 (0.96)
Impairment, n (%)	0 (0.0)
**Left Ankle Dorsiflexion ROM z-score, mean (SD)**	-0.43 (1.49)
Impairment, n (%)	2 (22.2)
**Right Ankle Dorsiflexion ROM z-score, mean (SD)**	-0.01 (1.59)
Impairment, n (%)	2 (25.0)
**Tumor Location: Tibia or Fibula (Lower)**	
**PPT z-score, mean (SD)**	0.55 (0.17)
Impairment, n (%)	0 (0.0)
**TUG time z-score, mean (SD)**	0.57 (1.10)
Impairment, n (%)	0 (0.0)
**BOT z-score, mean (SD)**	N/A
Impairment, n (%)	
**Grip strength, mean (SD)**	0.28 (0.86)
Impairment, n (%)	0 (0.0)
**Left Quadriceps strength z-score, mean (SD)**	-0.64 (0.83)
Impairment, n (%)	1 (33.3)
**Right Quadriceps strength z-score, mean (SD)**	-0.27 (1.17)
Impairment, n (%)	0 (0.0)
**Left Ankle Dorsiflexion strength z-score, mean (SD)**	-0.84 (0.56)
Impairment, n (%)	0 (0.0)
**Right Ankle Dorsiflexion strength z-score, mean (SD)**	0.51 (0.78)
Impairment, n (%)	0 (0.0)
**Left Ankle Dorsiflexion ROM z-score, mean (SD)**	-1.40 (1.83)
Impairment, n (%)	2 (50.0)

**Right Ankle Dorsiflexion ROM z-score, mean (SD)**	-1.41 (1.78)
Impairment, n (%)	1 (25.0)

SD = standard deviation; PPT = physical performance test; TUG = timed up and go; BOT = Bruininks-Oseretsky test of motor proficiency; N/A = mean not evaluable due to missing data or low numbers

St. Jude - Confidential
